# PCDH20 inhibits esophageal squamous cell carcinoma proliferation and migration by suppression of the mitogen-activated protein kinase 9/AKT/β-catenin pathway

**DOI:** 10.3389/fonc.2022.937716

**Published:** 2022-09-28

**Authors:** Yijiao Ning, Chaoqun Deng, Chunhong Li, Weiyan Peng, Chun Yan, Jing Ran, Weihong Chen, Yujia Liu, Jiuyi Xia, Lin Ye, Zhengqiang Wei, Tingxiu Xiang

**Affiliations:** ^1^ Gastrointestinal Surgical Unit, The First Affiliated Hospital of Chongqing Medical University, Chongqing, China; ^2^ Department of Oncology, Suining Central Hospital, Suining, China; ^3^ Chongqing Key Laboratory of Molecular Oncology and Epigenetics, The First Affiliated Hospital of Chongqing Medical University, Chongqing, China; ^4^ Chongqing Key Laboratory of Translational Research for Cancer Metastasis and Individualized Treatment, Chongqing University Cancer Hospital, Chongqing, China

**Keywords:** Pcdh20, MAP3K9, ESCC, tumor suppressor gene, Akt/β-catenin

## Abstract

Aberrant protocadherins (PCDHs) expression trigger tumor invasion and metastasis. PCDH20 anti-tumor functions in various tumor have been identified. Tumor suppression is due to Wnt/β-catenin pathway antagonism and may be suppressed caused by PCDH20 downregulation through promotor methylation, whereas PCDH20 effects and regulation mechanism in esophageal squamous cell carcinoma (ESCC) remains elusive. We analyzed PCDH20 effects on ESCC and underlying action mechanisms for PCDH20. We test PCDH20 expression in ESCC tissues and cells by semi-quantitative PCR (RT-PCR) and q-PCR (real-time quantitative polymerase chain reaction). MSP (methylation-specific PCR) was carried out to assess the methylation of PCDH20 in ESCC cells and tissues. Anti-tumor effects of PCDH20 in vitro were assessed by clone formation assay, CCK8 assay, Transwell assay, and flow cytometry. Nude mice tumorigenicity was used to assess PCDH20 anti-tumor effect in vivo. Online database, qPCR, and Western blotting were used to identify the downregulation of MAP3K9 by PCDH20, associated with AKT/β-catenin signaling inactivation. We found that PCDH20 expression was dramatically attenuated in esophageal cancer tissues and cells, maybe due to promotor methylation, and ectopic PCDH20 expression suppressed ESCC malignant biological phenotypes. PCDH20 exerted anti-tumor effects by MAP3K9 downregulation, which suppressed AKT/β-catenin signaling in ESCC cells.

**Conclusion:**

PCDH20 was a tumor suppressor gene, which antagonized AKT/β-catenin signaling pathway in ESCC by decreasing MAP3K9.

## Introduction

It is estimated that there were more than 570,000 new cases of esophageal squamous cell carcinoma (ESCC) worldwide in 2020. China has the high incidence of esophageal cancer with a mortality rate of over 70%. More than 90% of the pathological types are defined as squamous cell carcinoma (1). The highly invasive and metastatic nature of advanced esophageal cancer leads to poor clinical outcomes, although positive surgical intervention referred (2, 3). Therefore, profound investigations are ongoing to mechanistically illustrated ESCC proliferative and metastatic regulation.

Considerable evidence suggests that inactivation of protocadherin (PCDH) may potentiate tumor cell metastasis (4). More than 80 PCDH family members have been identified, which is the largest subfamily of the calciosin superfamily (4). Multiple PCDHs are located in the 13q21 region and considered as anti-tumor genes are frequently downregulated caused by methylation (5–8), a common epigenetic modification, with promotor hypermethylation associated with downregulated and silenced tumor suppressor gene expression, resulting in tumorigenesis (9). PCDH8 acts as a tumor suppressor gene in breast (10), liver (11), kidney (12), bladder (13), and ESCC (14). PCDH9 is the anti-oncogene in glioma (15) and gastrointestinal cancer (16). PCDH10 suppressed pancreatic cancer cell (17). PCDH20 inhibited non-small cell lung cancer cell (18) and hepatocellular cancer cell (19) but frequently downregulated due to promotor hypermethylation; furthermore, we discovered distinctive PCDH20 expression in adult esophageal tissues and paired cancer tissues; however, the role of PCDH20 in ESCC has never been reported.

Aberrant activation of Wnt/β-catenin signaling is tumorigenic in ESCC (20), resulting in increased cancer cell stemness, metastasis, and tumor invasion (21). AKT, a serine/threonine protein kinase, known as Rac or PKB, playing a vital role in modulating cell proliferation, survive and apoptosis. Solid evidence has shown that abnormal AKT signaling activation is related to ESCC lymph node metastasis, indicating an unsatisfactory prognosis (22). Cross talk between AKT-related pathways and Wnt/β-catenin signaling has been identified. AKT and GSK3β are key regulators of Wnt/β-catenin, with activated AKT demonstrated to affect Wnt/β-catenin signaling by degradative phosphorylation of GSK3β on Ser9. The degradative phosphorylation results in a decreased level of the Axin/adenomatous polyposis coli (APC)/GSK3β destruction complex (DC), which promotes the deposition of β-catenin and resulting in tumor development (23–25). Mitogen-activated protein kinase 9 (MAP3K9) has been identified as an oncogene in human renal cancer cell (26). In goat mammary epithelial cells (GMECs), MAP3K9 can upregulate the expression of AKT (27); some reports indicate that activated AKT promotes the phosphorylation of GSK-3β on Ser9, thus activating the Wnt/β-catenin pathway (28).

In this study, for the first time, we revealed PCDH20 promotor hypermethylation in ESCC cell lines, ectopic PCDH20 expression suppressed ESCC cells malignant biological phenotype in vitro and in vivo. Furthermore, we discovered PCDH20 impeded AKT/β-catenin signaling via stabilizing DC mediated by MAP3K9 downregulation, which may pave the way for identifying potential molecular target based on a deeper understanding of the regulatory mechanism in ESCC.

## Materials and methods

### Cell and tumor samples

The esophageal carcinoma cells KYSE150 and EC109 were cultured with RPMI 1640 (Gibco BRL, Karlsruhe, Germany), 10% fetal bovine serum (FBS; Invitrogen, Carlsbad, CA, USA), 100 U/ml penicillin, and 100 mg/ml streptomycin (gibco-brl) 100 mg at 37°C, incubated with 5% CO2. DMEM medium (high glucose, HyClone, Logan, USA) containing 10% FBS were selected to culture HEK293T cells. We extract DNA and RNA from esophageal carcinoma tissue and adjacent tissues. These adjacent tissues and paired cancerous tissues are mainly taken from the General Surgery Department of the First Affiliated Hospital of Chongqing Medical University. Patients data were included in **Table 3**. The research has been validated by the First Affiliated Hospital of Chongqing Medical University Institutional Ethics Committees. (Approval number 2016-75)

### DNA and RNA isolation

Based on protocols DNA was isolated from esophageal carcinoma tissues and cells with use of DNAzol® Reagent (Invitrogen) and QIAamp® DNA Mini Kit (Qiagen, Hilden, Germany). RNA was extracted by utilizing TRIzol reagent (Invitrogen, Carlsbad, USA) from esophageal carcinoma cell lines and tissues (29). We stored all DNA and RNA samples in a -80°C refrigerator.

### Semi-quantitative PCR and qPCR

RNA was isolated by TRIzol (Invitrogen, Carlsbad, CA, USA) reagent. Promega GoScript ™ reverse transcriptase (Promega) was used to reverse transcribe (30). GeneAmp RNA PCR system (Applied Biosystems) and Go-Taq (Promega, Madison, WI, USA) were selected to perform RT-PCR on cDNA obtained from reverse transcription, using β-actin as an internal reference (30). Meanwhile, we used 7500 Real-Time PCR System (Applied Biosystems) and SYBR Green (Thermo Fisher Scientific) for qPCR experiments (29). GAPDH was used as a control. Primer sequences used are shown in **Table 1** (see at the end of manuscript).

**Table 1:** List of primers in research.

PCR primer	Sequence (5′-3′)	Product size (bp)	Annealing temperature (℃)
PCDH20-F	ACCAGCTACAGGAACCTGC	291bp	55
PCDH20-R	GTCTAGGGTCACGTACTGG		
PCDH20-F1	TCTACATCGTCCCAGGAGCA	144bp	
PCDH20-R1	GCGGGTAGTCCCTCGTTTAG		
MAP3K9-F	ATTGGGCTGTGCAGATTGC	197bp	
MAP3K9-R	CGCACTCATCTTGGTGGTTC		
GAPDH-F	GGAGTCAACGGATTTGGT	206bp	55
GAPDH-R	GTGATGGGATTTCCATTGAT		
PCDH20m1	TTCGGCGATTTGGTATTCGC	127bp	60
PCDH20m2	CTACAACTTATCGAAATCGCG		
PCDH20u1	GTGTTTGGTGATTTGGTATTTGT	131bp	58
PCDH20u2	AATACTACAACTTATCAAAATCACA		

### DNA demethylation treatment

Esophageal carcinoma cells were cultured with 10 μmol/L 5-Aza-CdR (Sigma-Aldrich, St. Louis, MO, USA) for 3 days then treated with 100-nM histone deacetylase inhibitor TSA (trichostatin A) (Cayman Chemical Co, Ann Arbor, MI, USA) for a day or they were treated with 10 μmol/L 5-Aza-CdR for 4 days. Isolated RNA from wild-type cancer cell, Aza treatment group, and Aza+TSA treatment group respectively following the instruction from previous literature (29). RT-PCR was performed after RNA isolation, and MSP (methylation-specific PCR) was carried out while DNA isolation was completed (31).

### Methylation-specific PCR and bisulfite treatment

After we extracted the genomic DNA from cell lines and tissues by utilizing QIAamp DNA Mini Kit (Qiagen, Hilden, Germany), we modified it with bisulfite using previously reported modification methods (32, 33). The PCDH20 primers for MSP are shown in **Table 1**. We confirmed that all the primers used were unable to amplify unbisulfited DNA in advance, then the PCR amplify products were electrophoretic on 2% agarose gels.

### Establishing stable cell lines

Esophageal carcinoma cells EC109 and KYSE150 cells were seeded in 6-well plates. After the cell cultures reached 70–80% of confluency, pcDNA3.1-PCDH20 plasmid was transfected into EC109 and KYSE150 cells with Lipofectamine 2000 reagent (Lipofectamine 2000 Reagent, Invitrogen, CA, USA); refer to the manufacturer’s instructions for processes. Then, cells were cultured in six-well plate for 2 days, then G418-resistant clones were picked after 14-day selection.

### Colony formation assay

Colony formation assays were performed as previously described (30). Stably expressed PCDH20 ESCC cells and vectors were cultured in 6-well plates with different densities with 10% serum medium until 14 days, G418 was added at the same time. The number of surviving clones were stained with gentian violet then photographed with microscope and counted colonies over 50 cells.

### Cell proliferation assay

The cell viability was assessed by (CCK8, Promega) as previously described (30). Stably expressing PCDH20 cells or vector were cultured in 200 μl of a 96-well plate (2,000 cells per well) with 10% FBS-contained 1,640 for 24, 48, and 72h. The cells were co-cultured in 100 μl of serum-free 1640 containing 10 μl CCK8 at 37°C for 2h. Microplate reader (Multiskan MK3, Thermo Fisher Scientific, Schwerte, Germany) was used to measure absorbance at 450 nm.

### Cell cycle and apoptosis analyze

Cell cycle arrest and apoptosis were assayed by flow cytometry as described previously (30). Cells were digested with trypsin first, 70% ethanol ice-cold in advance was used to fix samples overnight, cells were treated with RNase A (Sigma-Aldrich, USA) at 5 mg/ml, stained with iodide in sodium propionate, and analyzed by flow cytometry (FACSCalibur instrument and CELLQUEST software, Becton Dickinson). Propidium iodide (PI) and Annexin V–fluorescein isothiocyanate (FITC; BD Pharmingen) were used in apoptosis detection. Quest software (BD Biosciences, San Jose, CA, USA) was used to analyze the result of apoptosis and cell cycle.

### Transwell migration and invasion assay

Transwell assays were carried out as described previously (34). Cell migration experiment was carried out in transwell chamber. Esophageal carcinoma cells stably expressing PCDH20 and empty vectors (2 × 104) were washed in serum-free medium and cultured in 24-well plate transwell filter. Lower chambers were filled with 800 μl culture medium containing 10% FBS. Cells incubated in 5% CO2 at 37℃ for 2 days, fixed with 4% paraformaldehyde for 30 min then stained with crystal violet. Cells that had not migrated or invaded through the pores were removed. Photographing representative images after erasing upper chamber cells.

### Tumorigenicity in xenograft nude mice model

The process of animal experiment abides by Chongqing Medical University Experimental Animal Center norm [approval number:2016-75]. ESCC cell EC109 (1 × 107) was suspended in 100 μl of phosphate-buffered saline (PBS). Cells were subcutaneously injected into the lower back of mice (n = 4). The length and width of the tumor were measured with vernier calipers each 5 days for a total of 28 days. Tumor volume = length × width2 × 0.52

### Immunohistochemistry

IHC was performed as previously described (35). Immunohistochemistry was carried out by utilizing the UltraSensitive TM SP Kit (Maixin-Bio, Fujian, China) following the protocols. Dewaxed sections from nude mice tumor tissues were rehydrated, antigen retrieval and antibody blocked, then incubated in 4℃ for 18h with primary antibody against 1:100 dilution Ki67(#ARG53222, Arigo)and PCDH20 (1:25 dilution, # NBP2-93484, Novus Biologicals Inc). Paraffin sections were treated with second antibodies. Diaminobenzidine (DAB, ZSGB-BIO, ZLI-9018) was used to stain the sections.

### Western blot

RIPA lysis buffer was used to extract total protein (Thermo Fisher Scientific, Waltham, MA, USA). The protein concentration was determined by BCA protein detection kit (Pierce, Rockford, IL, USA), after 10% gel electrophoresis the protein was transferred to PVDF (Bio-Rad, Hercules, CA, USA) membrane. Western blotting is carried out as previous described (29), the primary antibodies were used as follows: PCDH20 (# NBP2-93484, Novus Biologicals Inc), active β-catenin (#19807, Cell Signaling Technology), total β-catenin (# sc-7983, Santa Cruz Biotechnology), total GSK3β (#9315, Cell Signaling Technology), phosphor-GSK3β (# 9323, Cell Signaling Technology), P27 (#3686, Cell Signaling Technology), P21 (# sc-6246, Santa Cruz Biotechnology), cyclinD1 (# sc-8396, Santa Cruz Biotechnology), BCL-XL (# sc-8392, Santa Cruz Biotechnology), BCL-2 (#2870, Cell Signaling Technology), cleaved caspase-3 (#9664, Cell Signaling Technology), caspase-3 (#9662, Cell Signaling Technology) Bax(# sc-7480, Santa Cruz Biotechnology), Vimentin (#5741, Cell Signaling Technology),β-actin (#sc8432, Santa Cruz Biotechnology) and E-cadherin (#14472, Cell Signaling Technology), cleaved PARP (#5625, Cell Signaling Technology), and PARP (#9542, Cell Signaling Technology).

### Co-immunoprecipitation

The cells used for immunoprecipitation detection were washed with PBS twice and lysed with lytic buffer (Beyotime, P0013). After centrifugation(14,000g, 10 min, 4°C), the total protein and the primary antibody were rotated and incubated overnight at 4°C. Protein G-agarose beads (PROTGA-RO, Sigma-Aldrich) were added and incubated at 4°C for 2 h. After centrifugation, they were washed with lytic solution for 3 times. The magnetic beads adsorbing immune complexes were boiled with loading buffer for Western Blot.

### Statistical analysis

Statistical analyze was performed by SPSS (22.0 software). All the experiment results were evaluated with Fisher’s exact test, the χ2 test, and Two-tailed Student’s t-tests. All the p-values were less than 0.05 and the difference is statistically significant.

## Results

### Promoter methylation contributes to silenced expression of PCDH20 in ESCC

**Figure 1:**
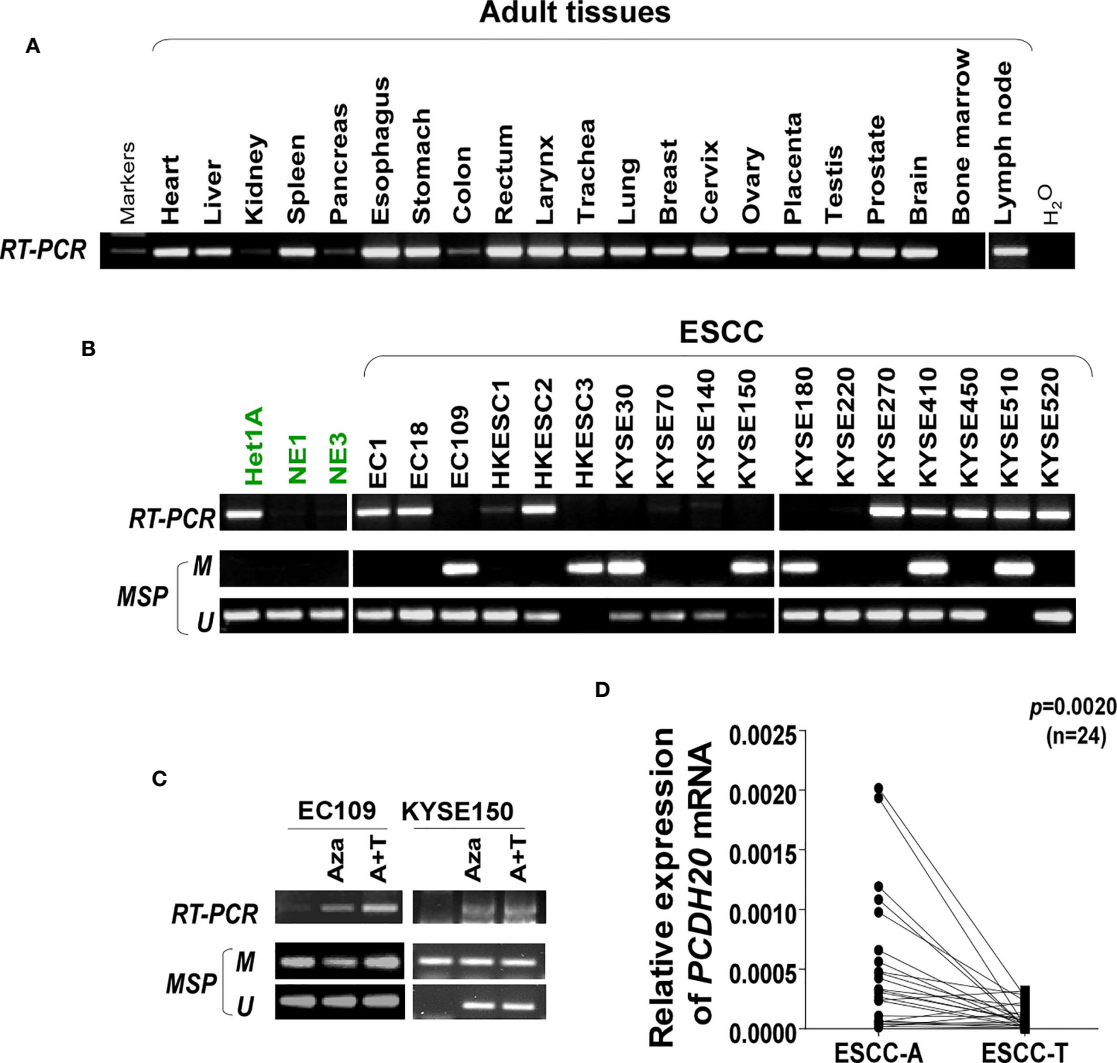
Expression and methylation of PCDH20 in esophageal squamous cell carcinoma tissues. **(A)** Expression of PCDH20 in normal tissues analyzed by RT-PCR. **(B)** RT-PCR and MSP analysis of PCDH20 mRNA expression and promoter methylation in esophageal squamous cancer cell lines. PCDH20 promotor hypermethylation could be found in some PCDH20-null cell lines including EC109, HKESC3, KYSE30, KYSE150, KYSE180, KYSE410, and KYSE510. **(C)** RT-PCR and MSP analysis of demethylation with 10 μmol/L Aza for 4 days or 1-day 100 nM TSA after 3-day Aza treatment (A+T) restored PCDH20 expression in EC109 and KYSE150 cell lines. **(D)** Quantitative real-time PCR (qPCR) analysis of PCDH20 mRNA expression levels in paired tumor and adjacent tissues (n = 24, p = 0.0020). Data are represented as M ± SD.

**Figure 2:**
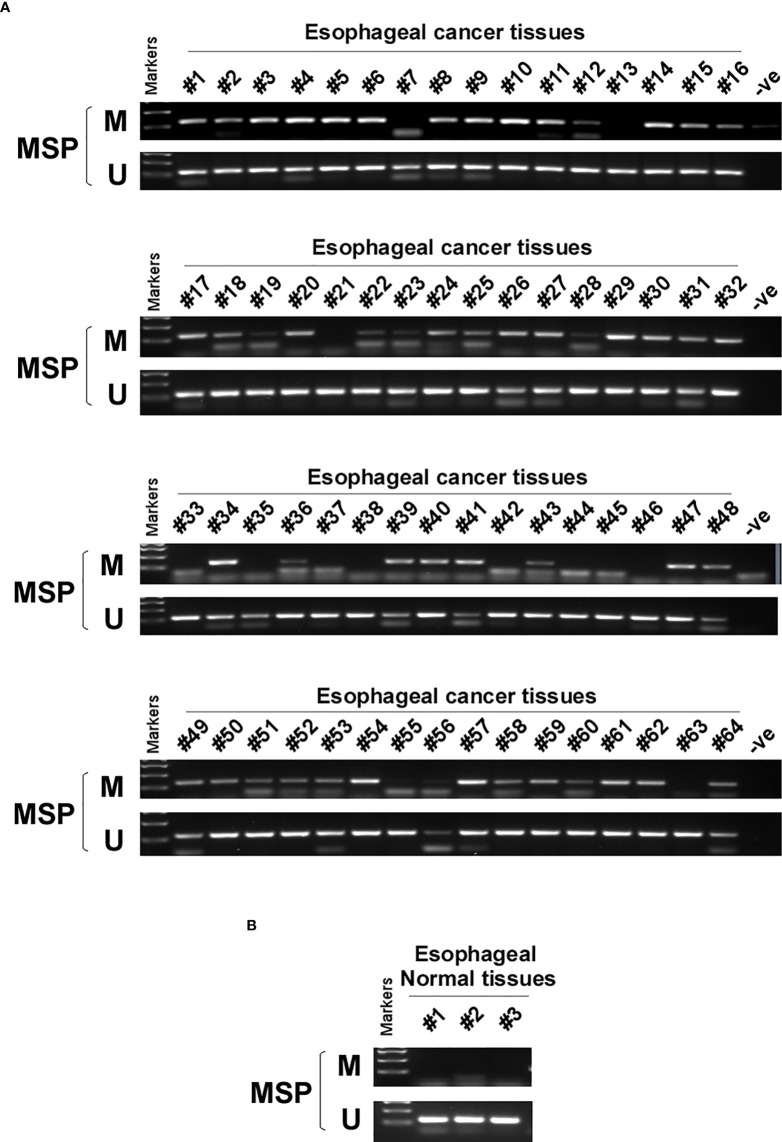
PCDH20 methylation status in normal esophageal tissue and ESCC tissue. **(A, B)** MSP was used to test PCDH20 promotor methylation status in ESCC tumor tissues and adjacent normal tissues.

DNA methylation is a common cause of tumor suppressor gene silencing. Previous studies have shown that promoter hypermethylation silences gene expression of PCDH20. We found that PCDH20 was silenced in many sorts of ESCC cell lines but ubiquitously expressed in normal tissues (**Figures 1A, B**), then we confirmed no PCDH20 mutations in EC109 and KYSE150 according to CCLE (Broad Institute Cancer Cell Line Encyclopedia). Therefore, we reasonably hypothesized that the low expression of PCDH20 in ESCC was related to promoter methylation. MSP demonstrated significant promoter CpG island methylation in some PCDH20-null cell lines (**Figure 1B**). In order to confirm that the methylation had a significant association with PCDH20 expression, demethylating drugs and HDAC inhibitor, Aza, and TSA (A+T) were used to treat EC109 and KYSE150 cell lines, resulting in modified expression of PCDH20. RT-PCR clarified that PCDH20 expression was restored inhibition of methylation (**Figure 1C**). The expression of PCDH20 mRNA in tumor tissues was reduced compared with that of adjacent tissues based on qPCR. (**Figure 1D**). MSP detected higher PCDH20 methylation in primary esophageal carcinoma tissues but no methylation in normal esophageal tissue samples (**Figures 2A, B** and **Table 2**; see at the end of manuscript). According to Fisher’s exact test, we found that methylation status of PCDH20 was not statistically associated with ESCC clinicopathological features (**Table 3**; see at the end of manuscript). Taken together, silenced expression of PCDH20 may be related to promotor methylation in esophageal carcinoma cells.

**Table 2:** PCDH20 promotor methylation status in ESCC and normal esophageal tissue.

Samples	PCDH20 promoter methylation status		Frequency
	methylation	unmethylation	
ESCC (n = 119)	77	42	77/119 (64.7%)
EN (n = 3)	0	3	0/3 (0%)

ESCC, esophageal squamous cell cancer; EN, esophageal normal tissue.

**Table 3:** Clinicopathological features of PCDH20 methylation in ESCC.

Characteristics	Number (n = 119)	PCDH20 methylation status		
		Methylated	Unmethylated	p-value
Age				
≤50	7	4 (57.1%)	3 (42.9%)	0.9801
>50	112	73 (65.2%)	39 (34.8%)	
Tumor size				
<3cm	33	19 (57.6%)	14 (42.4%)	0.844
≥3cm<5cm	62	39 (62.9%)	23 (37.1%)	
>5cm	17	11 (64.7%)	6 (35.3%)	
Unknown	7			
Lymph node metastasis				
Positive	39	25 (64.1%)	14 (35.9%)	0.575
Negative	80	47 (58.7%)	33 (41.3%)	
Pathological grade				
I	18	11 (61.1%)	7 (38.9%)	0.820
II	55	34 (61.8%)	21 (38.9%)	
III	43	29 (67.4%)	14 (32.6%)	
Unknown	3			

### PCDH20 overexpression inhibits colony formation and proliferation of ESCC cells

**Figure 3:**
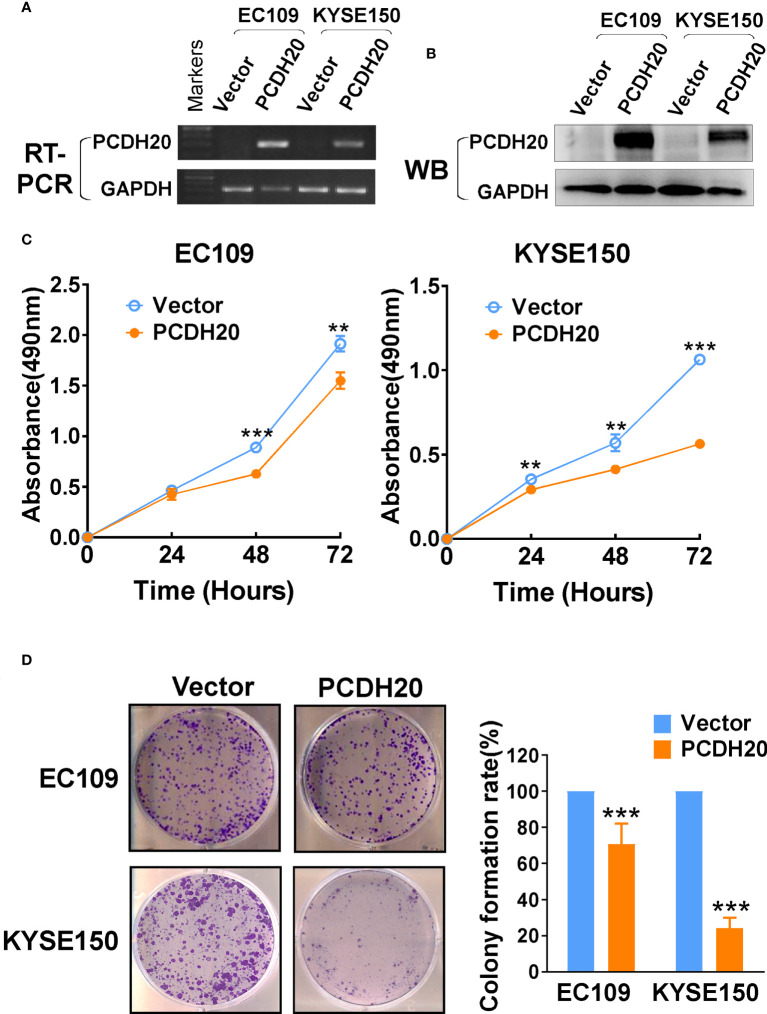
Ectopic expression of PCDH20 inhibited proliferation of ESCC cell lines. **(A, B)** RT-PCR and Western blot analysis of ectopic PCDH20 expressions in EC109 and KYSE150. **(C)** The effect of PCDH20 on cell viability was assessed by the CCK8 assay, wild-type EC109 and KYSE150 were used as controls. **p < 0.01, ***p < 0.001. Colony formation assay was used to assess the effect of PCDH20 on proliferation of EC109 and KYSE150. **(D)** Colony formation representative images and histogram statistics. All experiments were performed triplicate. **p < 0.01, ***p < 0.001. All the data were represented as M ± SD.

In order to investigate the anti-tumor effect of PCDH20 on esophageal carcinoma cells. PCDH20 was overexpressed in EC109 and KYSE150. Confirmation was carried out by RT-PCR and Western blotting (**Figures 3A, B**). CCK8 assays demonstrated the ectopic expression of PCDH20 in KYSE150 and EC109 cell lines to significantly decreased cell viability at 48 and 72h (**Figure 3C**). To assess the effects on cell proliferation, colony formation assays were performed, which demonstrated significant reductions in the number of colonies after overexpression of PCDH20 (**Figure 3D**). These results illustrated that PCDH20 suppressed proliferation of esophageal carcinoma cells.

### PCDH20 promotes cell cycle arrest in G1 phase and promotes ESCC cells apoptosis

**Figure 4:**
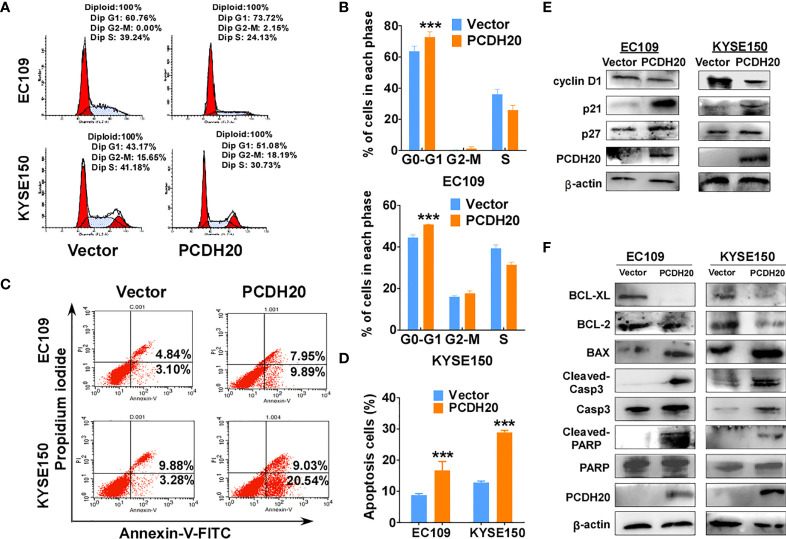
Overexpression of PCDH20 induced G1/G2 cell cycle arrest and promoted cell apoptosis of ESCC cells. **(A, B)** Flow cytometry analysis of the effect of PCDH20 on the cell cycle in PCDH20-overexpressing EC109 and KYSE150 cells. Wild-type EC109 and KYSE150 were used as controls. Left, flow cytometry plots. Right, histogram statistics of cell cycle variations. ***p < 0.001. **(C, D)** Flow cytometry analysis of the proportion of apoptotic cells in control (vector transfected) and ectopic PCDH20 expressing EC109 and KYSE150. Left, flow cytometry plots. Right, histogram statistics of the proportion of apoptotic cells. ***p < 0.001. Data above are represented as M ± SD. **(E)** Antibodies reactive with cell cycle related proteins; cyclin D1, P21, and P27 were used for western blot. **(F)** Antibodies reactive with apoptosis-related proteins; BCL-XL, BCL-2, Bax, cleaved-caspase 3, and cleaved-PARP were used for Western blotting.

Because PCDH20 had a significant inhibitory effect on the proliferation of esophageal carcinoma cells, we assessed effects on the apoptosis and cell cycle by utilizing flow cytometry, PCDH20 overexpression resulted in G1 phase cellular accumulation (**Figures 4A, B**) and increased esophageal cancer cells apoptosis (**Figures 4C, D**). By Western blotting, expression of cyclin D1 was significantly decreased, whereas P21 and P27 were upregulated in PCDH20-overexpressed cell lines (**Figure 4E**). In addition, the levels of Bcl-xl and Bcl-2 were downregulated, whereas the expressions of the pro-apoptotic proteins; Bax, cleaved-caspase-PARP; and cleaved-caspase-3 were upregulated in PCDH20 transfected cells (**Figure 4F**). These results showed that PCDH20 suppressed esophageal cancer cells proliferation mainly by promoting cell cycle arrest and apoptosis.

### Ectopic PCDH20 expression inhibits esophageal carcinoma cell migration, invasion, and EMT

**Figure 5:**
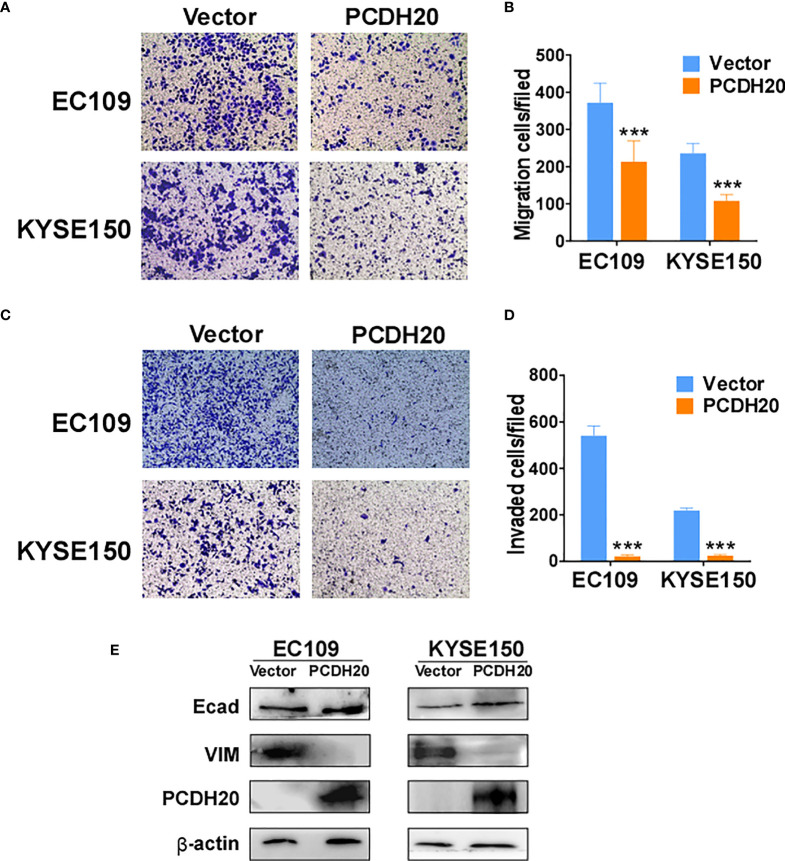
PCDH20 overexpression inhibited ESCC cell migration and invasion. **(A, B)** Left, Representative image of the transwell assay. Right, number of migrating cells in control (vector transfected) and PCDH20-expressing EC109 and KYSE150. ***p < 0.001. **(C, D)** Left, representative image of transwell invasion assay; right, number of invaded cells in control and PCDH20-expressing EC109 and KYSE150 cells. ***p < 0.001. Data above are represented as M ± SD. **(E)** The levels of E-cadherin and Vimentin in control (vector transfected) and PCDH20-expressing EC109 and KYSE150 cell lines by Western blotting.

PCDH20 functions as a calcium-coated protein that facilitates cellular adhesion and metastasis of tumor cells. Transwell assay results demonstrated that PCDH20 inhibited migration and invasion in esophageal cancer cells (**Figures 5A–D**). By Western blotting, PCDH20 overexpression increased levels of E-cadherin, whereas the level of vimentin was decreased (**Figure 5E**). These results demonstrated that PCDH20 dramatically weaken esophageal cancer cells migration and invasion abilities.

### PCDH20 inhibits ESCC growth *in vivo*

**Figure 6:**
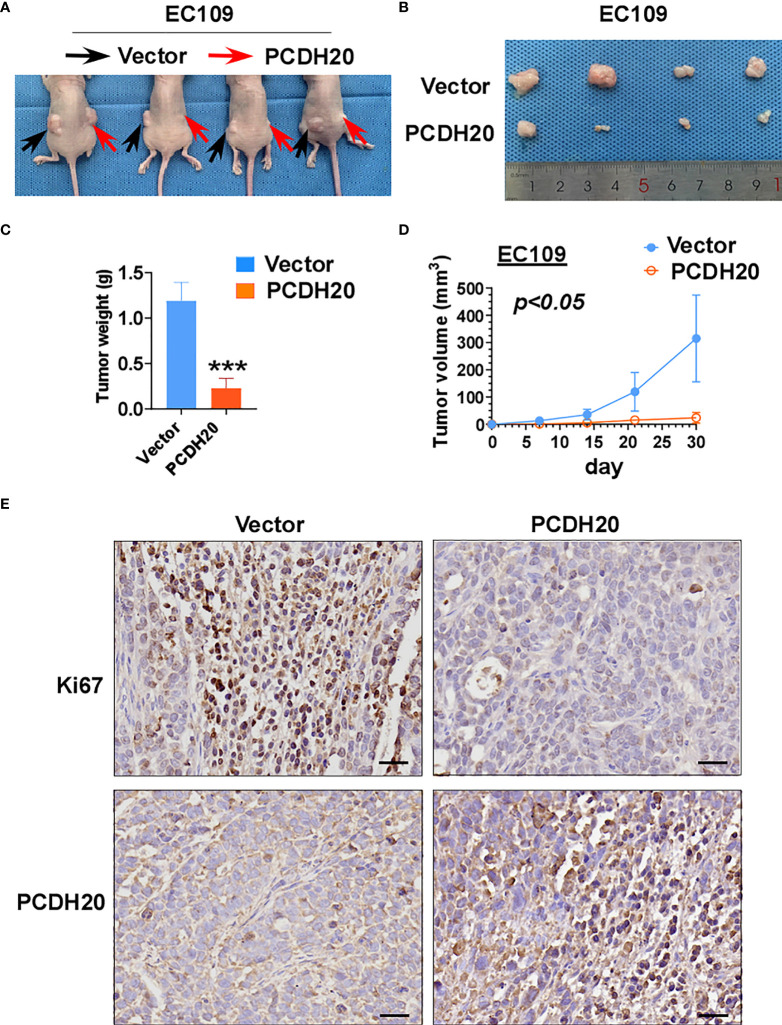
Overexpression of PCDH20 inhibited ESCC cells in vivo. **(A)** Representative image of nude mice tumorigenicity (n = 4). **(B)** Naked eye overview of tumor (n = 4). **(C, D)** Tumor weight and volume variation during 30 days in vector and PCDH20-overexpressing group ***p < 0.001. **(E)** Representative images of immunohistochemistry results of Ki67 and PCDH20 expression in nude mice tumor. (Magnification, ×400).

To further clarify the effect of PCDH20 on ESCC in vivo, we built a xenograft model in immunodeficient mice. We found that compared with the control group, the weight and volume of tumor decreased significantly in cells with ectopic expressing PCDH20 (**Figures 6A–D**). Ki67 is an index indicating the cancer cell proliferation potential. PCDH20 has obvious inhibitory effect on tumor proliferation in vivo, so we carried out IHC to assess Ki67 variation in PCDH20-overexpressing cell compared with vector group (**Figure 6E**).

### PCDH20 suppresses invasion and migration of ESCC by inhibiting MAP3K9 and Wnt/β-catenin pathways

**Figure 7:**
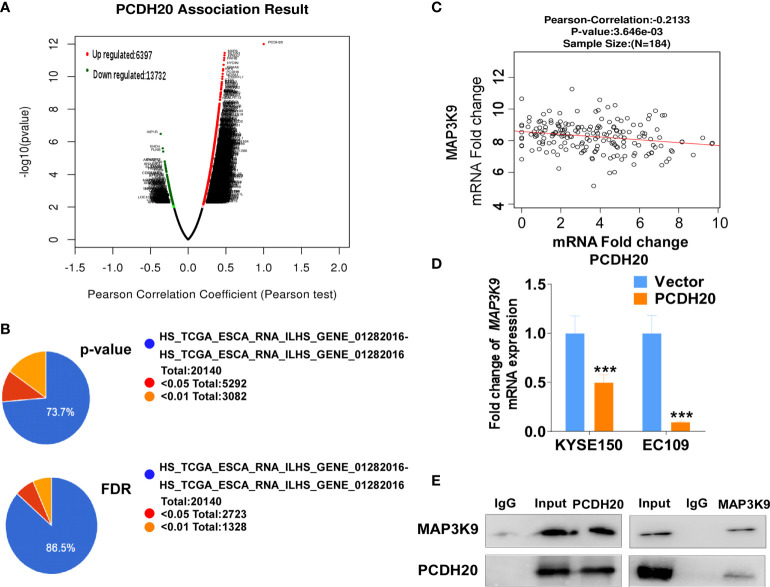
PCDH20 downregulated MAP3K9 expression. **(A)** PCDH20 association result in online database based on Pearson test. **(B) **The PCDH20-related genes were screened according to the original and adjusted statistic significant P-values. **(C)** Results of Pearson-Correlation between PCDH20 and MAP3K9 in database (p < 0.01). **(D)** Q-PCR showed decreased MAP3K9 mRNA expression after PCDH20 restoration ***p < 0.001. **(E)** Co-immunoprecipitation analysis of PCDH20 and MAP3K9.

**Figure 8:**
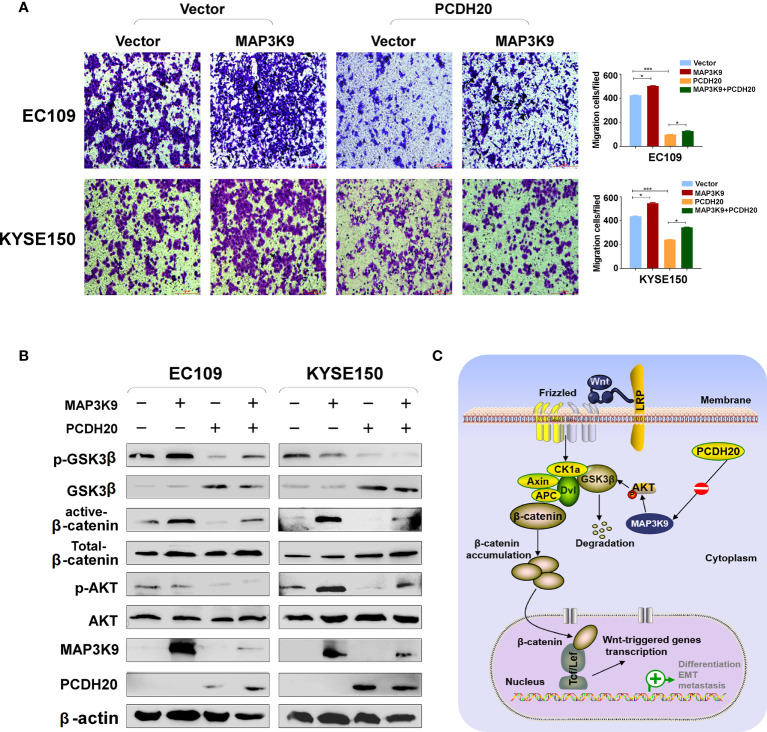
PCDH20 inhibited Wnt/β-catenin Pathway in ESCC by downregulating MAP3K9 expression. **(A)** Representative image and histogram statistics of the effect of MAP3K9 overexpression on migration of EC109 and KYSE150 cells. *p < 0.05; ***p < 0.001. **(B)** The protein levels of PCDH20 in EC109 and KYSE150 cells transfected with MAP3K9 and control (vector transfected). Antibodies were used, which were reactive with GSK-3β, p-GSK-3β, active-β catenin, total-β catenin, p-AKT, AKT, and PCDH20. β-actin was used as a control. **(C)** Diagram for PCDH20 modulated MAP3K9 to antagonist Wnt/β-catenin signaling by stabilizing β-catenin destruction complex.

PCDH20 association genes were analyzed in online database (http://www.linkedomics.org/), 20,129 in total related genes were identified, including 6,397 upregulated genes and 13,732 downregulated ones according Pearson test, summary result is obtained after adjustment p-value screening (**Figures 7A, B**). Dysregulation of canonical Wnt signaling pathway has been reported involved in ESCC progression. MAP3K9, as a MAPK, has been reportedly involved in regulation of Wnt/β-catenin signaling (36). A negative correlation between PCDH20 and MAP3K9 was shown in database (**Figure 7C**). In order to further confirm the result, we performed qPCR to verify the inhibitory effect of PCDH20 on MAP3K9. Results indicated that the expression of MAP3K9 was attenuated in PCDH20-overexpressing cell lines (**Figure 7D**). Co-immunoprecipitation uncovered a physical interaction between PCDH20 and MAP3K9 (**Figure 7E**). To further investigate the effects of MAP3K9 and PCDH20 on invasion and metastasis, we transfected MAP3K9 into cell lines and obtained the stably cell lines with expressing PCDH20. Transwell assays showed enhanced migration of esophageal cancer cells transfected with MAP3K9. (**Figure 8A**). Taken together, PCDH20 abolished ability of invasion and migration in ESCC cells by inhibition of MAP3K9. Given the evidence that MAP3K9 activated the Akt pathway in goat mammary epithelial cells (37), we speculated the inhibitory effects of PCDH20 on ESCC cells were mediated by MAP3K9/Wnt/β-catenin pathway. By Western blotting, levels of Wnt/β-catenin downstream proteins were assessed in cells overexpressing PCDH20. Results showed reductions in p-AKT, p-GSK3β, and active-β-catenin, whereas GSK3β levels increased in PCDH20 overexpressing esophageal cancer cells. Furthermore, we transfected MAP3K9 into esophageal cancer cell lines that stably expressed PCDH20. The levels of p-GSK3β, p-AKT, and active-catenin were increased after overexpression of MAP3K9. Active β-catenin, p-AKT, and p-GSK3β levels were restored in MAP3K9 overexpressing esophageal cancer cells after simultaneous stable expression of PCDH20 (**Figure 8B**). Taken together, these results demonstrated that the PCDH20 inhibited migration of esophageal cancer cells by downregulation of MAP3K9 expression, which antagonized Wnt/β-catenin signaling pathway (**Figure 8C**).

## Discussion

**Figure 9:**
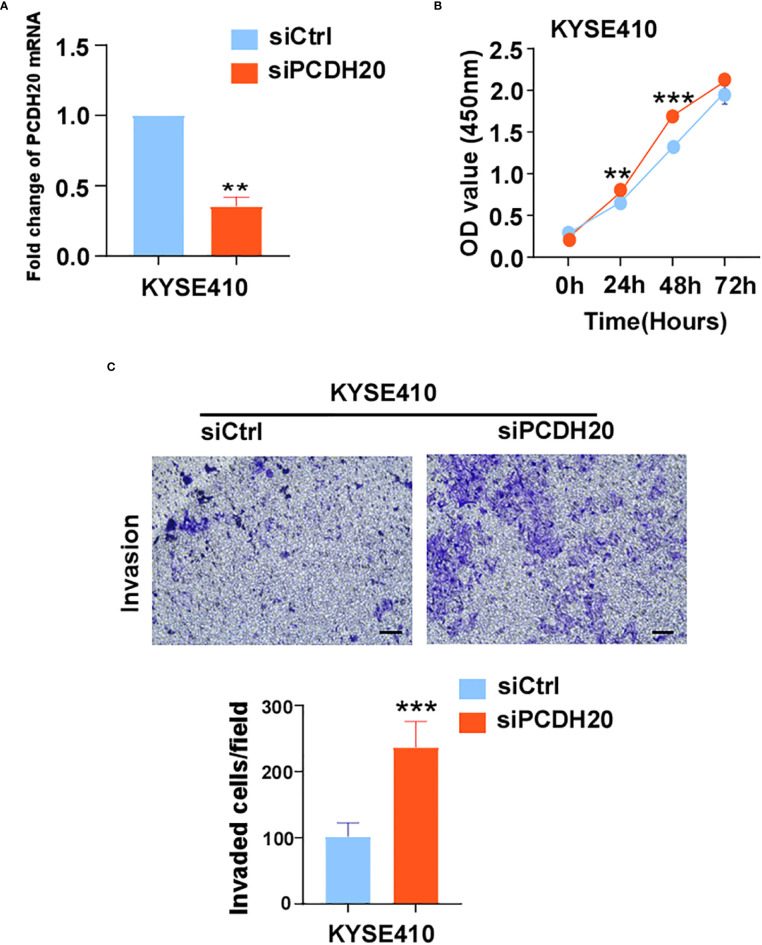
**(A)** Histograms of PCDH20 mRNA expression in negative control and siPCDH20 KYSE410 cells. **(B)** The effect of PCDH20 knock-down on KYSE410 cell viability measured by CCK8 assays. Red and blue curves represent siNC and siPCDH20 groups respectively **(C)** Representative images of invaded siNC and siPCDH20 transfected KYSE410 cells detected by Transwell assay. Histograms of number of invaded cells in siNC and siPCDH20 transfected KYSE410 cells. **p<0.01, ***p<0.001.

The known PCDH family contains approximately 80 members, which is the largest subfamily of the cadherin superfamily (38). PCDH δ, members of the PCDH family, are tumor suppressor genes and include PCDH8, PCDH9, PCDH10, and PCDH17 (39). The expression of PCDH20 is decreased or silenced in non-small cell lung cancer, hepatocellular carcinoma, and hypopharyngeal squamous cell carcinoma (18, 19, 37), but the function of PCDH20 in esophageal cancer cells is not well understood. CCK8 assay, colony formation assay, and xenograft model indicated PCDH20 abolish ESCC proliferative potential in vitro and in vivo. It is worth noting that knockdown of PCDH20 significantly promoted the proliferation and invasion of KYSE410 (**Figure 9**). Downregulation or silencing of tumor suppressor genes due to promoter methylation is a driver event in tumorigenesis (40, 41). We found that PCDH20 was widely expressed in a variety of normal cells but frequently silenced in esophageal carcinoma cell due to promoter methylation.

PCDH20 downregulates the activation of Wnt/β-catenin signaling in hepatocellular carcinoma and hypo-pharyngeal squamous cell carcinoma, whereas the mechanism of PCDH20 inhibiting ESCC remains unknown. Herein, we found that PCDH20 downregulated MAP3K9 expression in ESCC cell lines. MAP3K9, as an MAPK, activates the AKT-related pathway, which promotes degradative phosphorylation of GSK3β, induces degradation of the β-catenin degradation complex (actin/GSK3β/APC), stabilizing β-catenin in the cytoplasm, with abnormal cellular deposition of β-catenin resulting in tumor related events (42–45). Those data suggested that PCDH20 impeded GSK3β Ser9 phosphorylation by downregulating the expression of p-AKT, which ultimately resulted in β-catenin degradation and antagonizing Wnt/β-catenin pathway.

In summary, PCDH20 was frequently downregulated in ESCC cells by promoter methylation. PCDH20, as an anti-tumor gene in esophageal cancer cells, had a significant effect on the migration and invasion of ESCC cells, may mediated by inhibition of the MAP3K9/AKT/β-catenin pathway. These findings of our study provided insight into esophageal cancer metastasis and suggested new approaches to improved ESCC therapies.

## Data availability statement

The datasets presented in this study can be found in online repositories. The names of the repository/repositories and accession number(s) can be found in the article/supplementary material.

## Ethics statement

This study was reviewed and approved by Institutional Ethics Committees of the First Affiliated Hospital of Chongqing Medical University [approval number:2016-75]. The patients/participants provided their written informed consent to participate in this study. The animal study was reviewed and approved by Chongqing Medical University Experimental Animal Center norm [approval number:2016-75].

## Author contributions

TX: conception and design. YN, CL, CD: performed majority of experiments. WP, CY, WC: performed experiments and analyzed data. ZW, LY, YL, JX: collected samples. YN: drafted the manuscript. TX: reviewed data and manuscript. TX, YN: reviewed data and finalized the manuscript. All authors contributed to the article and approved the submitted version.

## Funding

This study was supported by National Natural Science Foundation of China (#82172619, #81872380, #82003202), Natural Science Foundation of Chongqing (2020ZYO13799, cstc2019jcyj-msxmX0861,2019ZX003).

## Conflict of interest

The authors declare that the research was conducted in the absence of any commercial or financial relationships that could be construed as a potential conflict of interest.

## Publisher’s note

All claims expressed in this article are solely those of the authors and do not necessarily represent those of their affiliated organizations, or those of the publisher, the editors and the reviewers. Any product that may be evaluated in this article, or claim that may be made by its manufacturer, is not guaranteed or endorsed by the publisher.
